# Impact of compulsory admission on treatment and outcome: A propensity score matched analysis

**DOI:** 10.1192/j.eurpsy.2022.4

**Published:** 2021-12-18

**Authors:** Andreas B. Hofmann, Hanna M. Schmid, Lena A. Hofmann, Vanessa Noboa, Erich Seifritz, Stefan Vetter, Stephan T. Egger

**Affiliations:** 1 Faculty of Medicine, Department of Psychiatry, Psychotherapy and Psychosomatics, Psychiatric University Hospital, University of Zurich, Zurich, Switzerland; 2 Faculty of Medicine, Department of Forensic Psychiatry, Psychiatric University Hospital, University of Zurich, Zurich, Switzerland; 3 Faculty of Medicine, San Francisco de Quito University, Quito, Ecuador; 4 Faculty of Medicine, Department of Psychiatry, University of Oviedo, Oviedo, Spain

**Keywords:** Coercion, compulsory admission, HoNOS, involuntary hospitalization, readmission

## Abstract

**Background:**

Despite multiple ethical issues and little evidence of their efficacy, compulsory admission and treatment are still common psychiatric practice. Therefore, we aimed to assess potential differences in treatment and outcome between voluntarily and compulsorily admitted patients.

**Methods:**

We extracted clinical data from inpatients treated in an academic hospital in Zurich, Switzerland between January 1, 2013 and December 31, 2019. Observation time started upon the first admission and ended after a one-year follow-up after the last discharge. Several sociodemographic and clinical characteristics, including Health of the Nation Outcome Scales (HoNOS) scores, were retrospectively obtained. We then identified risk factors of compulsory admission using logistic regression in order to perform a widely balanced propensity score matching. Altogether, we compared 4,570 compulsorily and 4,570 voluntarily admitted propensity score-matched patients. Multiple differences between these groups concerning received treatment, coercive measures, clinical parameters, and service use outcomes were detected.

**Results:**

Upon discharge, compulsorily admitted patients reached a similar HoNOS sum score in a significantly shorter duration of treatment. They were more often admitted for crisis interventions, were prescribed less pharmacologic treatment, and received fewer therapies. During the follow-up, voluntarily admitted patients were readmitted more often, while the time to readmission did not differ.

**Conclusions:**

Under narrowly set circumstances, compulsory admissions might be helpful to avert and relieve exacerbations of severe psychiatric disorders.

## Introduction

The detention and compulsory hospital admission for treatment are intended and regulated for psychiatric disorders [1]. While raising legal and ethical concerns, it is also a socially desired and regulated practice [1–3]. In most European countries, the legal framework of involuntary hospitalization usually requires the presence of a psychiatric disorder coupled with either self-harm or danger for others and the need for care [4], whenever less restrictive (e.g., ambulatory or day-clinic) treatment options are not feasible. Compulsory admission and treatment infringe on personal autonomy, they stigmatize both the patient and psychiatric practices and can be detrimental [4,5]. Previous studies have detected several risk factors for involuntary admission. These are not limited to sociodemographic and clinical characteristics of the patients but encompass several external conditions such as national legislation, community structure, or mental health resources [6]. Identified sociodemographic factors related to higher rates of compulsory admission are gender (male), civil status (unmarried, widow, single, or divorced), employment status (unemployed or welfare recipients), background, and ethnicity (immigrants and minorities) [7,8]. Age is related to the risk of compulsory admissions, although under different circumstances [8–11]. Organic, psychotic, and bipolar disorders are related to a higher rate of compulsory admissions [10–14], while affective and substance use disorders are related to a higher rate of voluntary admissions [12]. Several accompanying clinical features, such as aggression, disruptive behavior, suicidal thoughts, and cognitive impairment, have been considered risk factors. Previous involuntary hospitalizations, police involvement, and referral by on-call physicians are also related to a higher number of compulsory admissions [9]. Countries with higher healthcare spending and more inpatient beds also have higher rates of involuntary psychiatric hospitalizations [15].

Compulsory admission orders affect the patients’ autonomy and disturb the therapeutic relationship (and shared decision-making) [16]. Therapists have to reconcile the therapeutic needs and preferences of the patient, their wellbeing, and the reasons that lead to the compulsory admission itself [17,18]. Furthermore, they also affect the patients’ willingness and cooperation, to the extent that coercion or force might be required to treat the patient, directly hampering the therapeutic process and potentially diminishing the efficacy of the interventions [19]. Affected patients hold differing views regarding the need and success of compulsory admission orders [20,21]. The use of coercive measures, on the other hand, is ubiquitously disapproved and recognized as potentially traumatizing [22]. The risk factors, diagnoses, and clinical profiles related to a compulsory admission order and the involuntary treatment itself are determinants for treatment selection, and therefore outcomes. Despite its profound personal, therapeutic, ethical, and legal implications, compulsory admission order outcomes have not been thoroughly explored, which impedes a proper risk–benefit assessment of involuntary hospitalization as an intervention [18,19,23,24]. Therefore, we aim to explore factors associated with compulsory admission and, using propensity score matching, to assess the impact of compulsory admission on treatment, outcome, and service use. The latter is of critical importance due to the restriction of patients’ freedom and autonomy, requiring ethical reasoning and research on the benefits of involuntary hospitalization (and coercive measures) beyond security aspects.

## Methods

### Study setting and legal regulations

The Department of Psychiatry, Psychotherapy, and Psychosomatics, as part of the Psychiatric University Hospital of Zurich, is responsible for the psychiatric inpatient treatment of adult patients in the City of Zurich, Switzerland, and its surroundings, with a catchment area of approximately 500,000 inhabitants. The Canton of Zurich provides around 55 psychiatrists and 80 psychiatric beds per 100,000 inhabitants. The number of psychiatrists is above, and the number of psychiatric beds is marginally below the Swiss mean. Nevertheless, both rank higher than other Organisation for Economic Cooperation and Development (OECD) Countries [25–28]. Furthermore, the number of compulsory admissions in the Canton of Zurich is 2.2 per 1,000 inhabitants, which is high compared to national and international benchmarks [15,29].

The Swiss Civil Code and the Law for Protection of Children and Adults regulate the issue of a compulsory admission order and involuntary admission to a psychiatric institution [30,31]. Admission orders can be issued by either the competent authority or any practicing physician and, unless prolonged by the competent authority, run out after 6 weeks. The patient is entitled to demand their discharge and appeal the compulsory admission order in court at any time. Compulsory admission orders require a “mental disorder, mental disability or serious self-neglect if the needed treatment or care cannot be provided otherwise” *(Article 426 Swiss Civil Code).* In contrast to other countries, impending danger to the patients themselves or others is not essential for a compulsory admission order. However, it is required in the case of a compulsory retention order for voluntarily admitted patients *(Article 427 Swiss Civil Code).* Compulsory retention orders last up to 72 hours, during which they must be confirmed by the competent authority or an independent board-certified psychiatrist; otherwise, the patient must be discharged. Compulsory measures like isolation, restraint, or forced medication are usually used only in the presence of severe and imminent danger to patients themselves or others. They are regulated for psychiatric emergencies (*Article 435 Swiss Civil Code*). Under certain circumstances, forced treatment may electively be ordered to avert impending or repetitive complications resulting from non-treatment (*Article 434 Swiss Civil Code*).

### Study design, data sources

Our study retrospectively analyzed electronic health records of all first admissions and discharges from our hospital between January 1, 2013, and December 31, 2019. To record service use one year after discharge, we extended the collection period until December 31, 2020. We extracted routine clinical data from electronic health records for the present study. The Ethics Committee of the Canton of Zurich authorized the use of the anonymized data for research and publication purposes (BASEC: 2018-01906).

We used sociodemographic, clinical, and service use variables for the present analysis. Sociodemographic variables included age, gender, civil and educational status, German language proficiency, and migration status. We used the main treatment diagnoses according to the WHO-ICD-10 criteria. We used the Clinical Global Impression (CGI) scale and the Health of the Nation Outcome Scales (HoNOS) for the clinical evaluation. In addition, we extracted the pharmacologic and nonpharmacologic treatments prescribed during the hospitalization from the clinical records (Information Box 1). Service use variables included the type of admission, change of legal status during hospitalization, duration of treatment, type of discharge (i.e., regular or irregular in case of discharge against medical advice, court decision, death, or suicide of inpatients), and same hospital readmissions within one year after discharge.

**Table:** **Information Box 1.** Description of pharmacologic and nonpharmacologic treatments prescribed during hospitalization.

Treatment	Description
Pharmacologic	It refers to the prescription of medication for the treatment of a given condition or disorder. Pharmacologic treatments were classified according to the primary indication of the drug prescribed.
Crisis intervention	Crisis intervention is an immediate and short-term response to mental, emotional, and behavioral distress due to a psychiatric disorder. It responds to the patients’ needs; it provides emotional relief and dispensation from everyday duties.
Counseling	Counseling provides professional guidance, utilizing psychological methods. Its primary purpose is to restore the patients’ autonomy and ability to cope with everyday life.
Observation	Observation refers to the time used to observe and psychopathological symptoms and behavior in the case of a suspected or questioned diagnosis and when treatment effects (or side effects) are expected to occur.
Psychotherapy (single)	Psychotherapy in single sessions refers to the use of a structured psychotherapeutic approach to treat a specific condition or disorder. It uses specific psychological techniques that go beyond counseling or psychoeducation.
Psychotherapy (group)	Psychotherapy in a group session refers to a structured and manualized intervention delivered to patients with a common condition or disorder. It uses psychological group techniques and goes beyond educational or informative purposes.
Occupational therapy	Occupational Therapy refers to the use of self-care, art, work, sport, and play activities. Its primary purpose is to balance the patients’ daily activities, restore their autonomy, and help them cope with everyday life.

We classified the treatment diagnosis upon discharge in nine diagnostic groups according to the WHO-ICD-10 categories [32], in order to obtain representative and sufficiently large groups of patients: Dementia (F00–F04), Neurocognitive Disorders (F0X, X denotes the remaining categories), Alcohol Use Disorder (F10), Substance Use Disorders (F11–F19), Schizophrenia Spectrum Disorders (F2), Mania and Bipolar Disorder (F30–F31), Major Depressive Disorder (F3X), Anxiety and Stress-Related Disorders (F4–F5), and Personality Disorders (F6). Furthermore, we recorded the presence of comorbid alcohol and substance use (F10-F19) and personality disorders (F6).

The CGI Scales and the HoNOS were rated upon admission and discharge. The CGI is an easily applicable measurement instrument to assess severity (CGI-S) and improvement or deterioration during hospitalization (CGI-I). CGI-S is rated on a seven-point Likert scale from 1 (“normal”) to 7 (“extremely ill”). The CGI-I evaluates changes in comparison to the previous CGI evaluation. It ranges from 1 (“very much improved”) to 7 (“very much worse”), whereby a score of 4 indicates no change [33,34]. The HoNOS is a measurement instrument used to assess the severity of psychiatric disorders in 12 different domains covering behavior, symptomatology, impairment, and psychosocial functioning. Each item is rated on a five-point Likert scale from 0 (“no problem”) to 4 (“severe to very severe problem”). We evaluated the HoNOS at scale level (i.e., sum score ranging from 0 to 48) and item level [35–38]. We considered HoNOS Items rated three or four as clinically significant and as an integral part of the patients’ care plan [38].

### Statistical analysis

According to the principle of independence, the analysis only included the first admission between January 1, 2013, and December 31, 2019. Descriptive statistics (mean, standard deviation, median, interquartile range—IQR, and percentages) were used to characterize the whole sample according to their admission status (i.e., voluntary vs. compulsory). The propensity score represents the probability of individual cases to be compulsory admitted, conditional on their observed characteristics. Using logistic regression, we determined the relationship between sociodemographic and clinical characteristics and compulsory admission. Odds ratios (OR) were calculated with a 99% confidence interval (CI). Therefore, categorical variables were dichotomized, allowing to assess the risk associated with a single condition in contrast to all others not sharing this specific condition. The propensity score was calculated using logistic regression with the variables measured at admission. The model included sociodemographic, diagnostic, and clinical characteristics of the patients and the service use aspects (Information Box 2). Conditional on the propensity score, the distribution of observed baseline covariates will be similar between compulsory and voluntary admitted patients, allowing to assess the unbiased effect of compulsory admission order [39].

**Table:** **Information Box 2.** Variables (and their levels) used to calculate the propensity score.

Sociodemographic characteristics	Age, sex (male or female), marital status (single, married, divorced/separated, widowed, and others), education level (regular school, apprenticeship, or college/university), language proficiency (high or low), and migration status (Swiss citizen, migrant, nonsettled population as tourists, travelers, and refugees).
Clinical characteristics	Main psychiatric diagnosis (dementia, neurocognitive disorder, alcohol use disorder, substance use disorder, schizophrenia spectrum disorder, mania, bipolar disorder, major depressive disorder, anxiety- and stress-related disorder, and personality disorders) comorbid alcohol or substance use, and comorbid personality disorder.
Service use	Pathway to admission (emergency services, health professional, mental health professional, and others) and admission ward (closed, open, and facultative closed).
Psychometric and functional domains	Severity according to the CGI-S; HoNOS sum score; Number of HoNOS Items scored three or higher; single HoNOS Items scoring three or higher: Item 1: “Overactive, aggressive, disrupted or agitated behavior,” Item 2 “nonaccidental self-injury,” Item 3: “Problem drinking or drug-taking,” Item 4: “Cognitive problems,” Item 5: “Physical Illness or Disability,” Item 6: “Problems associated with hallucinations and delusions,” Item 7: “Problems with depressed Mood,” Item 8: “Other mental and behavioral problems,” Item 9: “Problems with relationships,” Item 10: “Problems with activities of daily living,” Item 11: “Problems with living conditions,” and Item 12: “Problems with occupation and activities.”

Each compulsorily admitted patient was matched in a 1:1 ratio to their unique nearest voluntarily admitted neighbor on the propensity score scale, with the smallest absolute, averaged propensity score distance across all included subjects [40,41]. If no matching pair was found, cases were excluded to guarantee similar distribution of variables in the secondary dataset. To assess the balance between the groups (before and after matching), we used the standardized mean difference (SMD) for continuous variables, the Chi-square (*χ*^2^) test for proportions, as well as propensity score distribution before and after matching (Table A1). We conducted an equivalence test for statistically different variables with a low effect size to determine whether the observed effect was smaller than our smallest effect size of interest (SD = 0.50). We chose a half standard deviation since it is consistently considered as a minimally important difference in health outcomes [42,43]. Two separated one-sided tests were performed to determine if the observed effect was larger than the lower bound (i.e., SD > −0.50) and less than the upper bound (i.e., SD < +0.50). Equivalence can be stated when the confidence interval rests within the equivalence boundaries [43–45].

All subsequent analyses were conducted with the propensity score matched sample. Variables measured at discharge were used to estimate the differences in treatment prescribed and outcomes between compulsorily and voluntarily admitted patients. We used Student’s *t*-test to assess differences in continuous variables and the Chi-square test (*χ*^2^) for differences in proportions. If assumptions about the distribution were not met, we additionally used an alternative nonparametric test (i.e., the Wilcoxon Signed-Rank Test). For changes in HoNOS sum scores, from admission to discharge, a single-factor independent group analysis of covariance (ANCOVA) was used to test for differences according to the admission status (i.e., voluntary vs. compulsory), thereby controlling for variability in scores upon admission. Two Kaplan–Meier time-to-event curves representing time to discharge (i.e., duration of treatment) and time to same hospital readmission were calculated; for testing the statistical significance, we used the log-rank *p*-value.

All tests of significance were two-tailed. Due to the large sample size, *p*-values less than .01 were considered significant. For significant results, SMD was used to evaluate effect sizes. For the analysis of the single HoNOS items, a Bonferroni correction for repeated measurements was performed. Because all remaining analyses were considered exploratory, no further correction for multiple comparisons was performed. Statistical analyses and figures were conducted using RStudio (2021.09.1 + 372); the statistical software R (4.1.2); and the R packages: tidyverse (1.3.1), TOSTER (0.3.4), MatchIt (4.3.1), survival (v 3.2–13), and survminer (0.4.9).

## Results

### Demographic and clinical characteristics of the study population

Between January 1, 2013, and December 31, 2019, 35,311 direct admissions and discharges occurred; 17,290 were individual first admissions. The mean age was 46.3 (19.4) years, with 49.0% (*n* = 8,474) females. Low German language proficiency occurred in 13.6% (*n* = 2,343) of the population. Almost one third of all admissions (30.4%, *n* = 5,250) were compulsory admissions. The most common diagnoses were major depressive disorder (29.2%, *n* = 5,042), anxiety and stress-related disorders (17.0%, *n* = 2,943), Schizophrenia spectrum disorders (15.8%, *n* = 2,729), and alcohol use disorders (11.2%, *n* = 1,935), accounting altogether for almost three quarters (73.2%, *n* = 12,649) of first admissions (Table 1).

**Table 1.** The sample’s demographic and diagnostic characteristics according to admission status (i.e., voluntary vs. compulsory), before and after propensity score matching.

	Whole sample			Matched pair sample		
Type of admission	Voluntary	Compulsory	SMD	Voluntary	Compulsory	SMD
	*n* = 12,040	*n* = 5,250		*n* = 4,570	*n* = 4,570	
Sociodemographic variables						
Age (years)	44.11 (17.44)	51.37 (22.48)	0.361	47.20 (20.19)	48.10 (21.00)	0.044
Sex			0.029			0.009
Female	5,953 (49.4)	2,521 (48.0)		2,201 (48.2)	2,180 (47.7)	
Male	6,087 (50.6)	2,729 (52.0)		2,369 (51.8)	2,390 (52.3)	
Civil status			0.187			0.064
Single	6,204 (51.5)	2,350 (44.8)		2,283 (50.0)	2,189 (47.9)	
Married	2,586 (21.5)	1,033 (19.7)		921 (20.2)	879 (19.2)	
Unmarried/other	3,250 (27.0)	1,867 (35.6)		1,366 (29.9)	1,502 (32.9)	
Education status			0.325			0.045
Regular school	5,739 (47.7)	3,320 (63.2)		1,119 (24.5)	1,059 (23.2)	
Apprenticeship	4,187 (34.8)	1,186 (22.6)		1,119 (24.5)	1,059 (23.2)	
College/university	2,114 (17.6)	744 (14.2)		593 (13.0)	653 (14.3)	
Residence status			0.174			0.007
Swiss citizens	9,091 (75.5)	3,869 (73.7)		3,343 (73.2)	3,355 (73.4)	
Migrants	2,403 (20.0)	922 (17.6)		887 (19.4)	875 (19.1)	
Tourists/other	546 (4.5)	459 (8.7)		340 (7.4)	340 (7.4)	
German proficiency			0.206			0.064
High	10,676 (88.7)	4,271 (81.4)		3,870 (84.7)	3,762 (82.3)	
Low	1,364 (11.3)	979 (18.6)		700 (15.3)	808 (17.7)	
Clinical variables						
Diagnosis			0.770			0.032
Dementia	136 (1.1)	296 (5.6)		133 (2.9)	133 (2.9)	
Neurocognitive disorder	352 (2.9)	734 (14.0)		328 (7.2)	328 (7.2)	
Alcohol use disorder	1,484 (12.3)	446 (8.5)		419 (9.2)	446 (9.8)	
Substance use disorder	754 (6.3)	229 (4.4)		256 (5.6)	229 (5.0)	
Schizophrenia spectrum disorder	1,438 (11.9)	1,296 (24.7)		1,209 (26.5)	1,209 (26.5)	
Mania and bipolar disorder	759 (6.3)	462 (8.8)		451 (9.9)	451 (9.9)	
Major depression	4,279 (35.5)	759 (14.5)		757 (16.6)	757 (16.6)	
Anxiety and stress-related disorders	2,227 (18.5)	714 (13.6)		712 (15.6)	712 (15.6)	
Personality disorders	611 (5.1)	314 (6.0)		305 (6.7)	305 (6.7)	
Psychiatric comorbidity						
Alcohol/substance use disorder	1,415 (11.8)	489 (9.3)	0.079	464 (10.2)	461 (10.1)	0.002
Personality disorder	1,290 (10.7)	326 (6.2)	0.162	368 (8.1)	323 (7.1)	0.037
Rating scales						
CGI-S	4.71 (1.03)	5.00 (1.08)	0.277	4.85 (1.07)	4.94 (1.09)	0.090
HoNOS sum score	18.15 (6.86)	21.25 (7.53)	0.431	20.40 (6.82)	20.88 (7.50)	0.070
HoNOS items > 3	3.43 (2.22)	4.36 (2.50)	0.392	4.00 (2.32)	4.23 (2.47)	0.095

Abbreviation: *SMD*, standardized mean difference.

### Relationship between sociodemographic and clinical variables with compulsory admission

Patients aged over 75 years (OR: 3.63, 99%CI: 3.19–4.13) and unmarried (OR: 1.49, 99%CI: 1.36–1.63) had an increased risk of being compulsorily admitted. Adults between 25 and 50 had a lower risk. Patients with low German language proficiency (OR: 1.79, 99%CI: 1.59–2.02), those who completed regular school education (OR: 1.89, 99%CI: 1.73–2.06), and nonresidential population (OR: 2.62, 99%CI: 1.70–2.33) were also at higher risk. Patients with dementia (OR: 5.23, 99%CI: 4.01–6.89), neurocognitive disorders (OR: 5.40, 99%CI: 4.55–6.43), or a schizophrenia spectrum disorder (OR: 2.42, 99%CI: 2.17–2.70) showed an increased risk for compulsory admission, while those with a major depressive disorder showed a decreased risk (OR: 0.31, 99%CI: 0.27–0.34). Patients showing “Aggressive and Disruptive Behavior” (HoNOS Item 1: OR: 5.23, 99%CI: 4.71–5.81), “Self-Harm” (Item 02: OR: 2.53, 99%CI: 2.21–2.89), “Cognitive problems” (Item 4: OR: 2.99, 99%CI: 2.71–3.29), “Hallucinations and Delusions” (Item 6: OR: 3.05, 99%CI: 2.75–3.38), and “Problems with Living Conditions” (Item 11: OR: 2.08, 99%CI: 1.90–2.29) had a higher probability of being compulsorily admitted, while those with “Depressed Mood” (HoNOS Item 7: OR: 0.50, 99%CI: 0.46–0.55) had a lower probability. For a full description of the variables, see Appendix.

### Propensity score matched paired sample

Using propensity score matching, we obtained a matched sample of 9,140 patients, 4,570 compulsorily and voluntarily admitted patients each. The mean age of the paired sample was 47.6 (20.6) years with 47.9% (*n* = 4,381) females. The more frequent diagnoses among compulsorily admitted patients were schizophrenia spectrum disorders (26.5%, *n* = 1,209), major depressive disorder (16.6%, *n* = 757), and anxiety and stress-related disorders (15.6%, *n* = 712). The balancing parameters of the matched pairs sample improved (Table A1). Upon admission, compulsorily admitted patients showed a higher HoNOS sum score (20.40 ± 6.82 vs. 20.88 ± 7.50; *t*(4,569) = 4.29, *p* < 0.001, *SMD* = 0.070) additional to a higher count of other clinically relevant items (4.00 ± 2.32 vs. 4.23 ± 2.47, *t*(4,569) = 4.65, *p* < 0.001, *SMD* = 0.095). However, the HoNOS sum score (*t*(8,949.45) = 20.63, *p* < 0.001) and the count of clinically relevant items (*t*(9,102.37) = 19.13, *p* < 0.001) were statistically equivalent. Severity gradings according to the CGI-S were (4.85 ± 1.07 vs. 4.94 ± 1.09; *t*(4,569) = 4.29, *p* < 0.001, *SMD* = 0.090) statistically different, and statistically equivalent (*t*(9,134.87) = 19.91, *p* < 0.001). No matched pair could be found for 680 (12.9%) of all compulsorily admitted patients. These were mostly patients aged 75 years (*n* = 515, 75.7%) or older and those diagnosed with dementia or neurocognitive disorders (*n* = 569, 83.7%) (see Appendix).

### Treatment, clinical outcomes, and service use parameters

**Figure 1.**
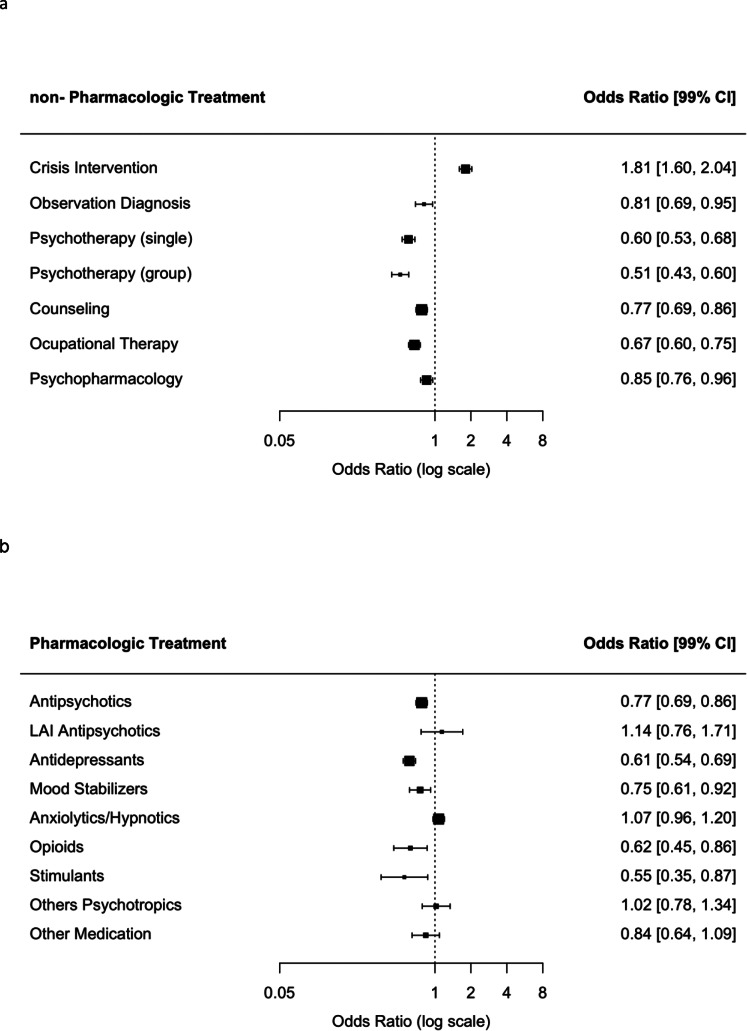
Odds ratios and 99% confidence intervals for treatment prescribed; the probability was calculated dichotomizing each variable. (A) Nonpharmacologic treatment. (B) Pharmacologic treatment. (C) Coercive treatment.

The duration of treatment was shorter for compulsorily admitted patients (24.44 ± 31.12 vs. 28.50 ± 28.70 days, *t*(4,569) = 6.56, *p* < 0.001). While hospitalized, the main treatment offered to compulsorily admitted patients was crisis intervention (64.6 vs. 76.7%, *χ*^2^(1) = 161.2, *p* < 0.001). Involuntarily hospitalized patients were less frequently assigned to other treatments, such as individual psychotherapy (31.5 vs. 21.6%, *χ*^2^(1) = 113.7, *p* < 0.001) or group psychotherapy (17.1 vs. 9.5%, *χ*^2^(1) = 112.3, *p* < 0.001), occupational therapies (41.3 vs. 32.3%, *χ*^2^(1) = 112.3, *p* < 0.001), with slightly lower rates of counseling (47.2 vs. 40.9%, *χ*^2^(1) = 36.8, *p* < 0.001), observation (13.6 vs. 11.3%, *χ*^2^(1) = 10.9, *p* < 0.001), and psychopharmacologic treatment (71.2 vs. 67.7%, *χ*^2^(1) = 12.4, *p* < 0.001) (Figure 1A). Psychopharmacologic therapy was overall less frequently prescribed to compulsorily admitted patients, especially antidepressants (35.5 vs. 25.1%, *χ*^2^(1) = 114.9, *p* < 0.001), mood stabilizers (8.8 vs. 6.7%, *χ*^2^(1) = 12.9, *p* < 0.001), stimulants (2.0 vs. 1.1%, *χ*^2^(1) = 11.2, *p* < 0.001), and opioids (3.7 vs. 2.3%, *χ*^2^(1) = 14.2, *p* < 0.001). Similar prescription rates were found for antipsychotics (49.6 vs. 48.2%, *χ*^2^(1) = 1.7, *p* = 0.18), LAI antipsychotics (1.7 vs. 1.9%, *χ*^2^(1) = 0.6, *p* = 0.43), anxiolytics/hypnotics (40.3 vs. 42.1%, *χ*^2^(1) = 2.74, *p* = 0.09), other psychotropics (4.8 vs. 4.0%, *χ*^2^(1) = 2.84, *p* = 0.09), and other medication (24.1 vs. 22.0%, *χ*^2^(1) = 5.7, *p* = 0.02) (Figure 1B). Compulsory patients were much more likely to be exposed to coercive measures, either as forced medication (2.3 vs. 8.7%, *χ*^2^(1) = 177.6, *p* < 0.001) or seclusion or restraint (2.7 vs. 9.3%, *χ*^2^(1) = 179.7, *p* < 0.001) (Figure 1C).

**Figure:**
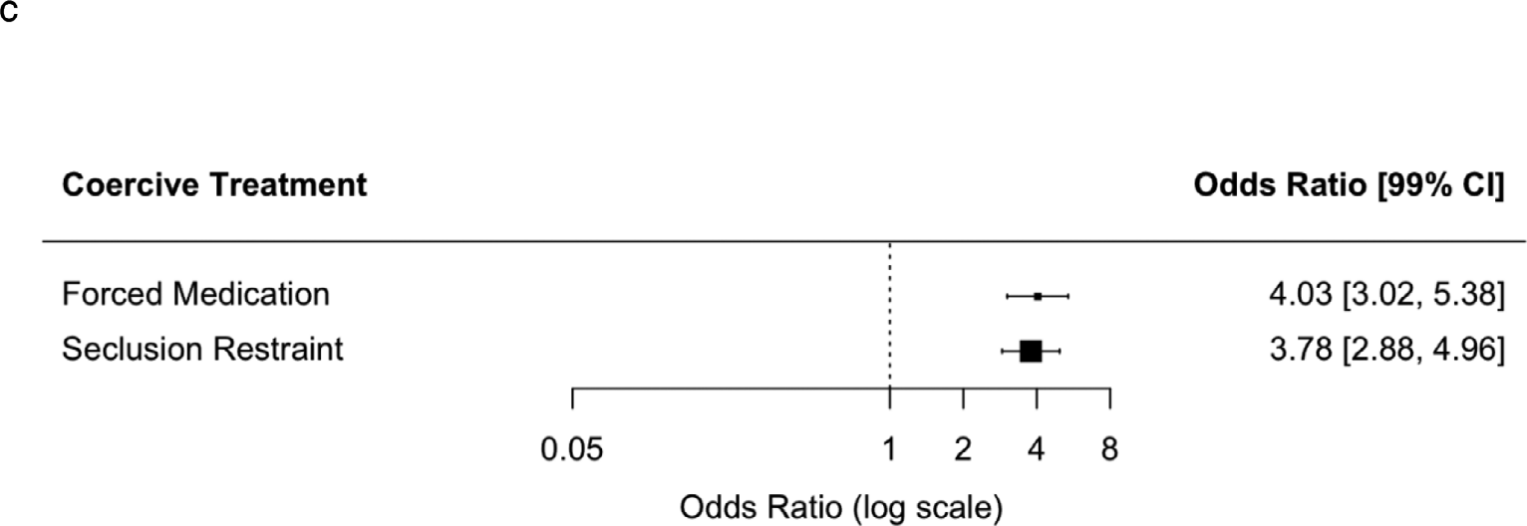

The HoNOS sum score improved for both groups from admission to discharge (*F*(3,13,000) = 960.3, *p* < 0.001), demonstrating that both groups experienced a significant improvement during hospitalization. Upon discharge, the HoNOS sum score (*p* = 0.23), and the number of clinically relevant items were similar (*p* = 0.57). Compulsorily admitted patients had a higher sum score difference (7.96 ± 7.33 vs. 8.63 ± 7.84, *t*(4,569) = 4.21, *p* < 0.001, *SMD* = 0.09), the difference was statistically equivalent (*t*(9,096.97) = 19.68, *p* < 0.001). Both groups had a similar percentage of change in the HoNOS sum score (41.5 ± 29.5% vs. 40.7 ± 39.6%, *t*(4,569) = 1.04, *p* = 0.29). According to CGI-I, compulsorily admitted patients experienced more improvement (2.57 ± 0.99 vs. 2.51 ± 0.99, *t*(4,569) = 4.65, *p* = 0.01; *SMD* = 0.05), although absolute differences remained small, and the results can be considered statistically equivalent (*t*(9,138) = 21.48, *p* < 0.001) (Table 2).

**Table 2.** The propensity score matched sample’s clinical and subsequent service use characteristics according to the admission status.

Type of admission	Voluntary	Compulsory			
	*n* = 4,570	*n* = 4,570	Statistic	*p*	SMD
Admission					
CGI-S	4.85 (1.07)	4.94 (1.09)	*t*(4,569) = 4.29	<0.001	0.090
HoNOS sum score	20.40 (6.82)	20.88 (7.50)	*t*(4,569) = 3.41	0.001	0.070
HoNOS items > 3	4.00 (2.32)	4.23 (2.47)	*t*(4,569) = 4.65	<0.001	0.095
Discharge					
CGI-I	2.57 (0.99)	2.51 (0.99)	*t*(4,569) = 2.55	0.01	0.053
HoNOS sum score	12.43 (7.08)	12.25 (7.43)	*t*(4,569) = 1.18	0.24	
HoNOS items > 3	1.53 (2.20)	1.56 (2.27)	*t*(4,569) = 0.56	0.57	
Clinical outcomes					
HoNOS difference score	7.96 (7.33)	8.63 (7.84)	*t*(4,569) = 4.21	<0.001	0.088
HoNOS percentage	41.52 (29.47)	40.72 (39.60)	*t*(4,569) = 1.04	0.27	
Service use					
Duration of treatment (days)	28.39 (28.52)	24.48 (30.92)	*t*(4,569) = 6.35	<0.001	0.131
Median (IQR)	21 (35)	14 (30)	*T* = 5,706,014, *z* = 4.49	<0.001	0.127
Readmission	1,750 (38.3)	1,564 (34.2)	*χ*^2^(1, 9,140) = 16.2	<0.001	0.085
Time to readmission (days)	96.95 (100.44)	100.22 (104.87)	*t*(326) = 1.050	0.29	
Median (IQR)	52 (152)	57 (160)	*T* = 25,203, *z* = −6.99	0.34	
Number of readmissions	2.30 (1.33)	2.22 (1.25)	*t*(3,304) = 1.95	0.05	
Type of discharge			*χ*^2^(1, 9,140) = 29.5	<0.001	0.114
Regular	4,400 (96.3)	4,289 (93.9)			
Irregular	161 (3.5)	260 (5.7)			
Death	8 (0.2)	19 (0.4)			
Suicide	1 (0.0)	2 (0.0)			
Change in willingness					
Compulsory retention	186 (4.2)	–			
Voluntary remain	–	1050 (23.0)			

Abbreviation: *SMD*, standardized mean difference.

**Figure 2.**
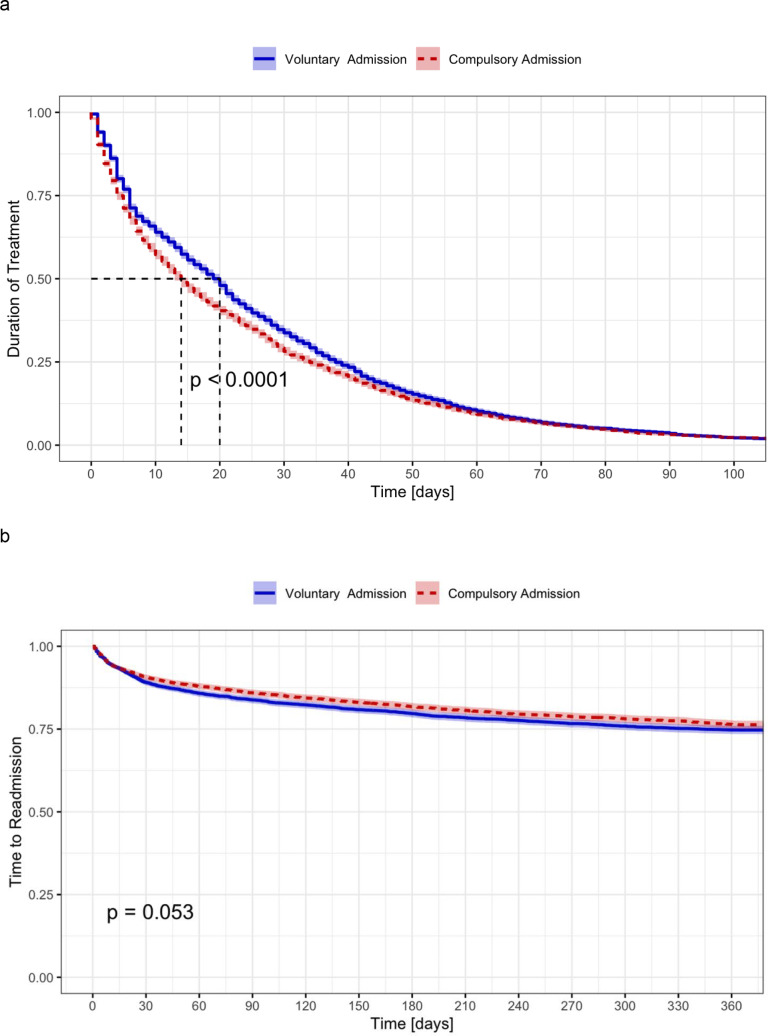
Kaplan-Meier time-to-event curves. (A) Duration of treatment. (B) Time to readmission.

The distribution of the duration of treatment was right-skewed for both groups (voluntary admissions: median: 21; IQR: 35 days; compulsory admissions: median: 14; IQR: 30 days), with compulsory admissions having a shorter length of stay (*T* = 5,706,014, *z* = 4.49, *p* < 0.001). The duration of treatment curve showed a significant difference between both groups. This difference becomes larger during the second and fourth weeks after admission (Figure 2A). The percentage of patients readmitted was higher for the voluntarily admitted patients (38.3 vs. 34.2%, *χ*^2^(1) = 43.34, *p* < 0.001). While the time to readmission did not differ (*p* = 0.34), the time to readmission curve (Figure 2B) was parallel for both groups. The number of readmissions was similar between both groups (2.30 ± 1.33 vs. 2.22 ± 1.24, *t*(3,304) = 1.95, *p* = 0.05). Although more irregular discharges were recorded for compulsory patients (3.2, *n* = 151 vs. 5.8%, *n* = 273, *χ*^2^(1) = 43.34, *p* < 0.001), almost a quarter (23%, *n* = 1,050) expressed the willingness to remain voluntarily in further inpatient treatment. On the other hand, 4.2% (*n* = 186) of all voluntarily admitted patients were retained against their will. Moreover, deaths (including suicides), although overall low, occurred almost three times more often in the compulsorily admitted group (0.2%, *n* = 9 vs. 0.5%, *n* = 21, *χ*^2^(1) = 4.04, *p* = 0.04) (Table 2).

## Discussion

The main finding of our study is that compulsorily admitted patients achieved a clinical improvement similar to voluntarily admitted patients in a shorter length of stay. During the 12 months following discharge, those initially voluntarily admitted had a higher readmission rate. This lower rate combined with the similar time to readmission suggests a robust and sustainable improvement in those compulsorily admitted. Furthermore, patients with a compulsory admission order were more frequently admitted for crisis intervention. Regarding their pharmacologic treatment, compulsorily admitted patients had an overall lower prescription rate, although they had higher rates of forced medication and seclusion or restraint. This finding is rather unsurprising since (imminent danger excluded) compulsory admission orders are legal requirements for coercive treatment [46].

The duration of treatment was shorter for compulsorily admitted patients, corroborating previous national findings [47]. This is opposed to international findings, where involuntary hospitalization has been related to a longer length of stay [48]. We consider this an effect of differences in legal stipulations between countries [47,49], with the Swiss legislation mandating to release the patients as soon as possible [30,31]. The shorter duration of treatment in compulsorily admitted patients cannot be attributed to previously identified sociodemographic or clinical characteristics [48] since these were balanced out through the propensity score matching.

Considering the overall lower prescription rates of pharmacologic and nonpharmacologic treatments in compulsorily admitted patients, outcomes and the shorter duration of treatment are not explained by the prescribed treatment. We consider that, in contrast to other medical specialties, psychiatric hospitalization is an intervention in itself, capable of averting danger and modifying the course of illness, thus resulting in a reduced burden of disease [50]. Furthermore, the improvement seems sustainable since the readmission rate to the same hospital is lower for compulsorily admitted patients, and the time to readmission is similar. However, the readmission rate should not be interpreted unconditionally as treatment success. A compulsory admission might have been such a disturbing experience [23], with the potential to elicit an avoidant behavior and a tendency to seek less help even if psychiatric inpatient care is needed [51]. Thus, it partially explains the lower readmission rate of compulsorily admitted patients within one year.

The clinical psychiatric evaluation requires sufficient observation time to verify the stability of an improvement [12,52,53]. Therefore, this might explain the differences in length of stay observed principally between the second and fourth weeks of treatment. Although an observation period of a few days might be insufficient for accurate clinical appraisal and sustainable intervention, a more extended treatment duration may indicate a more demanding clinical case [54–56]. Furthermore, as suggested by previous findings [14], compulsorily admitted patients with a longer length of stay usually chose to do this voluntarily when recommended to do so, leading to lower rates of discharge against medical advice as well as fewer appeals against the compulsory admission order.

We consider the observation time of one year after discharge appropriate since two-thirds of involuntary readmissions seem to occur within six months after the first hospitalization, an observation we also encountered in our population. Nonetheless, the same hospital readmission rate is a controversial measure for service use since the patients have the freedom to choose a specific institution and may not approach a hospital to which they were compulsorily admitted in the past [57]. In contrast, involuntary patients do not have this freedom of choice since the catchment areas determine the institution responsible for inpatient treatment. Therefore, changes in institutions are unlikely in these cases.

Regarding the risk factors for involuntary hospitalization, our results align with previous studies [10,58]. Older ages were associated with an increased risk of compulsory admission orders in our sample, while adults faced an overall lower risk. Older age might relate to the effects of aging, while younger age may be related to the peak of thought disorders [8–11]. Other cultural and social factors associated with an increased compulsory admission rate included low (German) language proficiency, lack of vocational/professional training, and psychotic symptomatology. Psychiatric diagnoses characterized by cognitive impairment (i.e., neurocognitive and neurodegenerative disorders) or thought disorders (schizophrenia, mania, and bipolar disorder) increased the risk for compulsory admission [8,46]. The clinical HoNOS profile of compulsorily admitted patients showed higher rates in domains relating to harm and danger, cognitive impairment, and psychotic symptoms [59].

The main strength of our study is the large clinical sample collected under the same legal framework over a long period [11]. When analyzing, interpreting, and comparing our results, the interplay between patient characteristics, local mental health services peculiarities, and legal regulations must be considered. They regulate and determine the patients’ access to treatment and directly influence therapeutic interventions [6,15,60–62]. In Switzerland, the rate of compulsory admission orders is high compared to other countries, suggesting a relatively low threshold for their use [11,62]. In contrast to previous findings, this might explain why danger or harm to oneself was also related to a compulsory admission order [10]. We consider propensity score matching a valid method to control confounding variables and reduce the potential bias by indication [63,64]. Using propensity score matching, we could balance the modifying effects of sociodemographic variables, diagnosis, and severity over treatment selections and, therefore, outcomes [65].

To reduce the possible flaws of propensity score matching, we selected the 1:1 ratio and unique nearest neighbor, a robust propensity score matching approach [41,66]. The balancing indices substantially improved after matching. Nonetheless, the clinical rating scales HoNOS and CGI-S still showed a statistically significant difference, although they had low effect size and were statistically equivalent [43–45]. We consider the statistically significant difference an artifact of the large sample size [67,68]. Unfortunately, matching was not feasible for the whole sample, with 12.9% (*n* = 680) of compulsorily admitted patients ending up without a counterpart, mainly comprising patients older than 75 years with a neurocognitive disorder. Due to the unique characteristics of this group and their influence on treatment selection, their exclusion led to a reduction in bias.

One main limitation of our study is the inability to deduce the individual’s perception of neither impaired personal integrity, nor legitimation and usefulness, as patients’ treatment satisfaction is prognostic for future involuntary admissions [20]. Nevertheless, we can infer that a large proportion of compulsorily admitted patients accept hospitalization as a reasonable option since almost a quarter of patients agrees to remain voluntarily in treatment, and irregular discharge is just two percentage points higher than in voluntarily admitted patients. Another point to consider is that patients suffering from critical, life-threatening medical conditions are usually transferred to a general hospital before being referred to a psychiatric institution (e.g., in case of severe intoxication or anorexia). Thus, the rate of deceased patients could be inaccurate, limiting comparability [24]. Finally, from our data, we cannot account for informal coercion or suggestion before or during treatment, though other research suggests its relevant influence [15,62,69].

In summation, the presence of danger to oneself or others in combination with factors that impair communication, collaboration, and bonding impede outpatient treatment. Thus, the lack of compliance and commitment to treatment undermines the efficacy of outpatient compulsory treatment orders [46,70]. In circumstances when the need for care is urgent, a compulsory admission order becomes an imminent alternative [59,71]. Our results show that compulsorily admitted patients are more likely to present harmful or dangerous behavior, coupled with impaired communication and bonding. They experience forced medication and seclusion or restraint more frequently. However, they show a robust clinical improvement within a shorter time than voluntary patients. The lower readmission rate coupled to similar time to readmission suggests a sustainable improvement. From our analysis, compulsory admission orders leading to involuntary hospitalization appear to be a meaningful intervention to reduce dangerous and harmful behavior. However, due to its ethical and legal implications, the threshold for such an enormous impairment of a person’s rights and freedom must be carefully outweighed. Therefore, both in routine clinical practice and future studies, the patients’ opinions and the notion of treatment have to be considered even more.

## Figures and Tables

**Figure A1.**
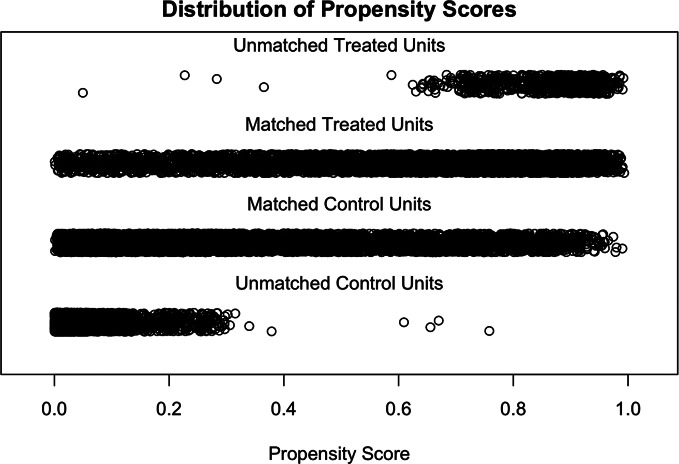
Propensity score distribution for the matched and nonmatched samples.

**Figure A2.**
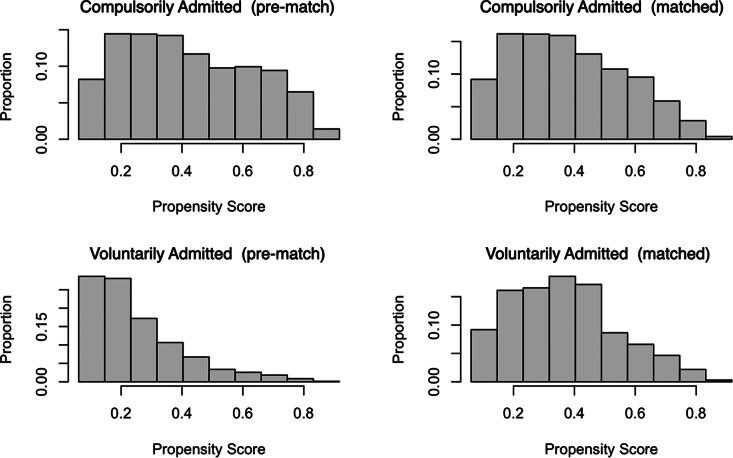
Pre- and post-matching propensity score distribution.

## Data Availability

The data supporting the findings of this study are available from the corresponding author upon reasonable request. The data are not publicly available due to privacy or ethical restrictions.

## Appendix

**Table A1.** Relation between the single variables and compulsory admission.

	Estimate	SE	*z*-Value	*p*-Value	OR (99% CI)
Sociodemographic characteristics					
Age					
16–24	−0.030	0.469	−0.653	0.51	0.97 (0.86–1.09)
25–39	−0.473	0.038	−12.40	<0.001	0.62 (0.56–0.69)
40–49	−0.360	0.045	−8.083	<0.001	0.70 (0.62–0.78)
50–64	−0.140	0.043	−3.275	0.001	0.87 (0.78–0.97)
65–74	0.464	0.062	7.477	<0.001	1.59 (1.35–1.86)
75 or older	1.289	0.049	25.88	<0.001	3.63 (3.19–4.13)
Sex					
Female	−0.057	0.033	−1.723	0.08	0.94 (0.87–1.03)
Marital status					
Single	−0.271	0.033	−8.174	<0.001	0.76 (0.70–0.83)
Married	−0.110	0.041	−2.678	0.007	0.90 (0.80–1.00)
Unmarried	0.400	0.035	11.32	<0.001	1.49 (1.36–1.63)
Migration status					
Swiss	−0.095	0.037	−2.527	0.01	0.91 (0.82–1.00)
Migrant	−0.157	0.042	−3.674	<0.001	0.85 (0.76–0.95)
Nonsettled population	0.701	0.656	10.69	<0.001	2.02 (1.70–2.39)
Educational level					
Regular school	0.636	0.033	18.73	<0.001	1.89 (1.73–2.06)
Apprenticeship	−0.603	0.038	−15.80	<0.001	0.55 (0.50–0.60)
College/university	−0.254	0.046	−5.503	<0.001	0.78 (0.69–0.87)
Language proficiency					
Low	0.584	0.045	12.81	<0.001	1.79 (1.59–2.02)
Clinical characteristics					
Main diagnosis					
Dementia	1.654	0.105	15.76	<0.001	5.23 (4.01–6.89)
Neurocognitive disorder	1.685	0.067	25.10	<0.001	5.40 (4.55–6.43)
Alcohol use disorder	−0.415	0.056	7.313	<0.001	0.66 (0.57–0.76)
Substance use disorder	−0.381	0.077	−4.936	<0.001	0.68 (0.56–0.83)
Schizophrenia spectrum disorder	0.882	0.042	20.71	<0.001	2.42 (2.17–2.70)
Mania and bipolar disorder	0.361	0.062	5.865	<0.001	1.43 (1.22–1.68)
Major depression	−1.182	0.0436	−27.11	<0.001	0.31 (0.27–0.34)
Anxiety and stress related disorders	−0.365	0.0466	−7.85	<0.001	0.69 (0.61–0.78)
Personality disorders	0.173	0.071	2.432	0.02	1.19 (0.99–1.43)
Comorbid alcohol or substance use disorder	−0.259	0.055	−4.699	<0.001	0.77 (0.67–0.89)
Comorbid personality disorder	−0.594	0.064	−9.244	<0.001	0.55 (0.47–0.65)
Psychometric and functional domains					
Item 1: “Overactive, aggressive, disrupted, or agitated behavior”	1.653	0.040	40.46	<0.001	5.23 (4.71–5.81)
Item 2: “Nonaccidental self-injury”	0.927	0.051	17.99	<0.001	2.53 (2.21–2.89)
Item 3: “Problem drinking or drug-taking”	−0.190	0.037	−5.136	<0.001	0.83 (0.75–0.91)
Item 4: “Cognitive problems”	1.095	0.037	29.16	<0.001	2.99 (2.71–3.29)
Item 5: “Physical illness or disability”	0.401	0.040	9.95	<0.001	1.49 (1.35–1.66)
Item 6: “Problems associated with hallucinations and delusions”	1.114	0.040	27.74	<0.001	3.05 (2.75–3.38)
Item 7: “Problems with depressed mood”	−0.687	0.034	−20.26	<0.001	0.50 (0.46–0.55)
Item 8: “Other mental and behavioral problems”	−0.142	0.035	−4.12	<0.001	0.87 (0.79–0.95)
Item 9: “Problems with relationships”	0.230	0.034	6.80	<0.001	1.26 (1.15–1.37)
Item 10: “Problems with activities of daily living”	0.397	0.034	11.79	<0.001	1.49 (1.36–1.62)
Item 11: “Problems with living conditions”	0.733	0.036	20.38	<0.001	2.08 (1.90–2.29)
Item 12: “Problems with occupation and activities”	0.170	0.034	4.97	<0.001	1.19 (1.09–1.29)

**Table A2.** Demographic and clinical characteristics of the sample without a matching pair.

	No match (*n* = 680)
Age (mean, SD)	73.35 (19.58)
Gender (percentage)	
Female	341 (50.1)
Civil status (percentage)	
Single	161 (23.7)
Married	154 (22.6)
Unmarried	365 (53.7)
Education status (percentage)	
Regular school	462 (67.9)
Apprenticeship	127 (18.7)
College/university	91 (13.4)
Residence status (percentage)	
Swiss citizens	514 (75.6)
Migrant	47 (6.9)
Tourist/other	171 (25.1)
German proficiency (percentage)	
Low	106 (18.8)
Main diagnosis	
Dementia	163 (24.0)
Neurocognitive disorder	406 (59.7)
Alcohol use disorder	–
Substance use disorder	11 (1.9)
Schizophrenia spectrum disorder	87 (12.8)
Mania and bipolar disorder	11 (1.6)
Major depression	2 (0.3)
Anxiety and stress related disorders	2 (0.3)
Personality disorders	9 (1.3)
Psychiatric comorbidity	
Comorbid alcohol or substance use disorder	28 (4.1)
Comorbid personality disorder	3 (0.4)
